# Mathematical analysis of hybrid mediated blood flow in stenosis narrow arteries

**DOI:** 10.1038/s41598-022-15117-6

**Published:** 2022-07-26

**Authors:** Azad Hussain, Lubna Sarwar, Aysha Rehman, Qasem Al Mdallal, Abdulrazak H. Almaliki, A. S. El-Shafay

**Affiliations:** 1 Department of Mathematics, University of Gujrat, Gujrat, 50700 Pakistan; 2 Department of Mathematical Sciences, UAE University, Alain, 15551 United Arab Emirates; 3 Department of Civil Engineering, College of Engineering, Taif University, P. O. Box 11099, Taif, 21944 Saudi Arabia; 4 Department of Mechanical Engineering, College of Engineering, Prince Sattam Bin Abdulaziz University, Alkharj, 16273 Saudi Arabia; 5 Mechanical Power Engineering Department, Faculty of Engineering, Mansoura University, Mansoura, 35516 Egypt

**Keywords:** Mathematics and computing, Nanoscience and technology

## Abstract

In this paper the behavior of flow of blood under stenosis suppositions is studied. Nanoparticles of Ag and Cu are being used with blood as base fluid. The problem governing equations are modeled into PDE’s, which are transformed into set of ODE’s with the help of useful similarity transformation. We investigated the solution numerically for various parameters on temperature and velocity distribution and shown in the form of tables and graphs. It is found that the velocity of blood increases while the temperature curve goes down by increasing the concentration of nanoparticles and also temperature curve decreases by increasing the values of gamma and Prandtl number. Furthermore, the calculated results shows that increment in flow parameter gamma caused an increase in velocity values. In the field of biomedicine, the important approach of nanotechnology is the use of nanoparticles in chemotherapy.

## Introduction

The mechanical investigation of blood flow through arterial stenosis allows some important aspects because of its significant medical and engineering applications. It is very normal to find the narrowing’s or plaques in the arterial system of humans, known as arterial stenosis. The normal pattern of flow of blood disturb through the stenosed artery. To filter waste products our kidneys require particular amount of flow of blood and that normal amount of rich-oxygen blood can’t reach our kidneys due to tapered arteries and this may cause high blood pressure and many injuries. Stenosis in arteries can cause death due to worsen over time. Ellahi et al.^1^ investigated the flow of blood through permeable walls stenosis and use perturbation method to find the solution of their problem. Haghighi et al.^2^ analyzed the blood flow behavior mathematically through constrict arteries and solved the problem by using finite difference method. Hussain et al.^3^ described the blood flow behavior through tapered arteries by assuming blood as non-Newtonian fluid and investigated the flow problem numerically. Sankar et al.^4^ analyzed the narrow surface of arteries and did the comparative study by assuming it as two fluid model and consequences shows that resistance to flow increases with the increase of stenosis width and height. Shah et al.^5^ discussed some new properties in the inquiry of nanoscience’s that highlight the nanoparticles concentration in blood flow and studied the consequences of different parameters like source, sink, clot size and stenosis height etc. Pokhrel et al.^6^ studied abnormal flow of blood through diseased artery by using Navier stoke equations and investigated pressure drop, the ratio of minimum, maximum shear stress. Many investigations^7–10^ have been considered to understand the effects of stenosed arteries on blood flow. Tanveer et al.^11^ considered non-Newtonian fluid behavior of blood and presented theoretical analysis in a microchannel. Some other arterial diseases also discussed by many researchers^12–14^.

In biological systems the nanoparticles characteristics such as surface shape, chemistry, size can be controlled to increase their function. These properties vary for every nanofluid depending upon base fluid and the tiny particles. Nanoparticles have more ability to conduct heat as compared to base fluid and also have more impact on the heat transfer enhancement. In biomedical applications a large number of nanoparticles have been improved and in treatment some of them have shown great potential, to activate the growth of blood vessels and imaging of diseases. They are also treated as drug carrying vehicles. Cu-Ag nanoparticles are the most impactful materials in nanoscience and nanomedicine. Ardahaie et al.^15^ studied the consequences of nanoparticles through stenosis and also investigated the magnetic effects in a porous blood artery. Nadeem and Ijaz^16^ presented the characteristics of flow of blood through curved channel stenosis and also discussed the behavior of various parameters like slip parameter, Darcy number, Prandtl number and nanoparticles volume fraction. Zaman^17^ analyzed the behavior of unsteady blood flow in a stenosed channel in the presence of copper and silver nanoparticles. By using curvilinear coordinates he developed the equation of momentum and energy. Tripathi et al.^18^ investigated the characteristics of unsteady flow of blood through stenosed artery with the addition of (copper, silver) nanoparticles by considering porous arterial wall. They computed the various results for wall shear stress, temperature, velocity, flow rate for particular height of stenosis. Many researchers^19–32^ studied the applications of nanoparticles.

In order to indicate that how addition of nanoparticles can be helpful in blood flow through arteries, a mathematical model is investigated. In our problem, the base fluid blood is Newtonian fluid and hybrid nanoparticles are added to it. The results of various parameters including blood flow parameter, Prandtl number and nanoparticles volume fraction are studied and their consequences on flow are presented in the form of tables and graphs. In our paper firstly the flow governing equations are analyzed and solution is attained numerically by using MATLAB bvp4c technique. Next, the consequences of different parameters have been shown by plotting the graphs. At the last the important results are given. Present study is applicable in different biomedical fields.

## Flow geometry and coordinate system

The conditions of abnormal blood flow due to stenosis are developed and become the reason for disease in artery. In under discussion problem we assumed that flow of blood reacts like steady, incompressible, two dimensional, viscous fluid through constrict artery of length $\frac{{L}_{0}}{2}$, where blood flow along $x{\text{-}}axis$ and $r{\text{-}}axis$ is perpendicular to the flow.

**Figure 1:**
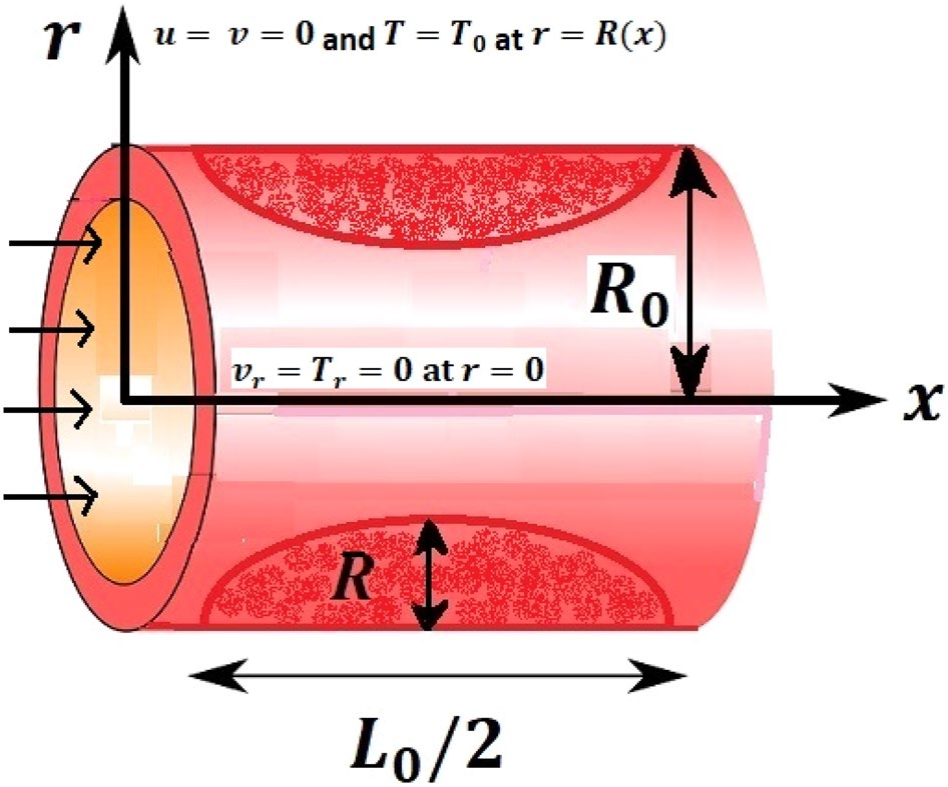
Geometrical structure of arterial stenosis.

The sketch of the problem as presented in Fig. 1, is described as Verma^33^, where blood flow through cosine shape constriction of artery having width ${2R}_{0}$ of unblocked area, radius of the blocked region of artery is $R\left(x\right)$ and the maximum height of constrict area is λ. The red dots shows the blockage in the artery and these blockage effect the blood flow. Stenosed region profile is selected as

1 $$ \begin{array}{ll}    R\left(x\right)={R}_{0}-\frac{\uplambda }{2}\left(1+\rm{cos}\left(\frac{4\pi x}{{L}_{0}}\right)\right), & \quad \quad {-\frac{{L}_{0}}{4}<x< \frac{{L}_{0}}{4}}  \\    \quad \quad \; {= {R}_{0}} & \quad \quad {Otherwise.}  \\   \end{array}  $$

## Governing equation and boundary conditions

The flow governing steady boundary layer equations for Newtonian hybrid nanofluid are defined as^34,35^:

2 $$\frac{\partial (ru)}{\partial x}+\frac{\partial (rv)}{\partial r}=0,$$

3 $$u\left(\frac{\partial }{\partial x}+v\frac{\partial }{\partial r}\right)u=\frac{{\upmu }_{hnf}}{{\uprho }_{hnf}}\frac{\partial }{r\partial r}\left(r\frac{\partial u}{\partial r}\right),$$

4 $$\left(u\frac{\partial }{\partial x}+v\frac{\partial }{\partial r}\right)T=\frac{{\mathrm{k}}_{hnf}}{{\left(\uprho {C}_{p}\right)}_{hnf}}\frac{\partial }{r\partial r}\left(r\frac{\partial T}{\partial r}\right),$$

together with boundary conditions

5 $$u= v=0 \;\; \mathrm{and} \;\;T={T}_{0} \;\;\mathrm{at} \;\;r=R\left(x\right),$$

$$\frac{\partial v}{\partial r}=0, \;\;\frac{\partial T}{\partial r}=0 \;\;\mathrm{ at} \;\;r=0.$$

Thermophysical properties of nanofluids are defined as follows^36^:

6 $$\left.\begin{array}{l}{\rho }_{nf}={\rho }_{f}\left(\left(1-\phi \right)+\phi \frac{{\rho }_{s}}{{\rho }_{f}}\right),\\ {\mu }_{nf}=\frac{{\mu }_{f}}{{\left(1-\phi \right)}^{2.5}},\\ {(\rho {C}_{p})}_{nf}={(\rho {C}_{p})}_{f}\left(\left(1-\phi \right)+\phi \frac{{(\rho {C}_{p})}_{s}}{{(\rho {C}_{p})}_{f}}\right),\\ \frac{{k}_{nf}}{{k}_{f}}=\frac{{k}_{s}+2{k}_{bf}-2\phi \left({k}_{bf}-{k}_{s}\right)}{{k}_{s}+2{k}_{bf}+\phi \left({k}_{bf}-{k}_{s}\right)}.\end{array}\right\}$$

7 $$\left.\begin{array}{l}{\rho }_{hnf}= \left(1-{\phi }_{2}\right)\left(\left(1-{\phi }_{1}\right){\rho }_{f}+{\phi }_{1}{\rho }_{{s}_{1}}\right)+{\phi }_{2}{\rho }_{{s}_{2}},\\ {\mu }_{hnf}=\frac{{\mu }_{f}}{{\left(1-{\phi }_{1}\right)}^{2.5}{\left(1-{\phi }_{2}\right)}^{2.5}},\\ {\left(\rho {C}_{p}\right)}_{hnf}=\left(1-{\phi }_{2}\right)\left(\left(1-{\phi }_{1}\right){\left(\rho {C}_{p}\right)}_{f}+{\phi }_{1}{\left(\rho {C}_{p}\right)}_{{s}_{1}}\right)+{\phi }_{2}{\left(\rho {C}_{p}\right)}_{{s}_{2}},\\ {k}_{hnf}={k}_{f}\frac{\left(\frac{{k}_{{s}_{2}}{\phi }_{2}+{k}_{{s}_{1}}{\phi }_{1}}{{\phi }_{1}+{\phi }_{2}}+2{k}_{f}\right)-2\left({\phi }_{1}+{\phi }_{2}\right)\left({k}_{f}-\frac{{k}_{{s}_{2}}{\phi }_{2}+{k}_{{s}_{1}}{\phi }_{1}}{{\phi }_{1}+{\phi }_{2}}\right)}{\left(\frac{{k}_{{s}_{2}}{\phi }_{2}+{k}_{{s}_{1}}{\phi }_{1}}{{\phi }_{1}+{\phi }_{2}}+2{k}_{f}\right)+\left({\phi }_{1}+{\phi }_{2}\right)\left({k}_{f}-\frac{{k}_{{s}_{2}}{\phi }_{2}+{k}_{{s}_{1}}{\phi }_{1}}{{\phi }_{1}+{\phi }_{2}}\right)},\end{array}\right\}$$

where ${\uprho }_{hnf}$ is the density, ${\upmu }_{hnf}$ is viscosity, ${k}_{f}$ and ${k}_{hnf}$ represents thermal conductivity of Cu-Ag nanoparticles and blood, ${(\uprho {C}_{p})}_{hnf}$ represents the heat capacity of fluid, which are described in Table 1. By introducing stream function $\psi$ The continuity Eq. (2) is satisfied for $u$ and $v$ such that

**Table 1:** Base fluid (blood) and Silver (Ag), Copper (Cu) nanoparticles experimental values^37^.

Material	Symbol	$\rho \;(\text{kg}/\text{m}^{3})$	${C}_{p}\; (\text{Jkg}^{-1}\text{K}^{-1})$	$k$ (${\text{Wm}^{-1}\text{K}}^{-1}$)
Blood	–	1050	3617	0.52
Silver	Ag	10,500	235	429
Copper	Cu	8933	385	400

8 $$u={r}^{-1}\frac{\partial \psi }{\partial r}, v={-r}^{-1}\frac{\partial \psi }{\partial x}.$$

Now Eqs. (3–4) becomes

9 $$\frac{1}{r}\frac{\partial \psi }{\partial r}\frac{\partial }{\partial x}\left(\frac{1}{r}\frac{\partial \psi }{\partial r}\right)-\frac{1}{r}\frac{\partial \psi }{\partial x}\frac{\partial }{\partial r}\left(\frac{1}{r}\frac{\partial \psi }{\partial x}\right)=\frac{{\upmu }_{hnf}}{{\uprho }_{hnf}}\frac{\partial }{r\partial r}\left(\frac{{\partial }^{2}\psi }{\partial {r}^{2}}-\frac{1}{r}\frac{\partial \psi }{\partial r}\right),$$

10 $$\left( \frac{1}{r}\frac{\partial \psi }{\partial r}\right)\frac{\partial T}{\partial x}-\left(\frac{1}{r}\frac{\partial \psi }{\partial x}\right)\frac{\partial T}{\partial r}=\frac{{\mathrm{k}}_{hnf}}{{\left(\uprho {C}_{p}\right)}_{hnf}}\frac{\partial }{r\partial r}\left(r\frac{\partial T}{\partial r}\right).$$

we utilize the following transformation to find the solution of Eqs. (7–8)

11 $$u=\frac{{u}_{0}x}{{L}_{0}}{F}^{{\prime}}\left(\eta \right), v=-\frac{R}{r}\sqrt{\frac{{u}_{0}{\nu }_{f}}{{L}_{0}}}F\left(\eta \right), \eta =\frac{{r}^{2}-{R}^{2}}{2R}\sqrt{\frac{{u}_{0}}{{{\nu }_{f}L}_{0}}} , \theta \left(\eta \right)=\frac{T-{T}_{0}}{{T}_{1}-{T}_{0}},$$

$$\psi =\sqrt{\frac{{u}_{0}{x}^{2}{\nu }_{f}}{{L}_{0}}}RF\left(\eta \right).$$

Setting ${x}=\frac{\tilde x}{{L}_{0}}$ and after using similarity transformation the Eqs. (9–10) finally takes the following form:

12 $$\frac{1}{{C}_{1}{C}_{2}}\left[\left(1+2\gamma \eta \right){F}^{{{\prime}}{{\prime}}{{\prime}}}+2\gamma {F}^{{{\prime}}{{\prime}}}\right]+F{F}^{{{\prime}}{{\prime}}}-{{F}^{{\prime}}}^{2}=0,$$

13 $$\begin{aligned} \mathrm{where }\,\,{C}_{1}={\left(1-{\phi }_{1}\right)}^{2.5}{\left(1-{\phi }_{2}\right)}^{2.5}, {C}_{2}=\left[\left(1-{\phi }_{2}\right)\left\{\left(1-{\phi }_{1}\right)+{\phi }_{1}\frac{{\rho }_{{s}_{1}}}{{\rho }_{f}}\right\}+{\phi }_{2}\frac{{\rho }_{{s}_{2}}}{{\rho }_{f}}\right]. \end{aligned}$$

14 $$\frac{1}{Pr {C}_{3}}\left[\left(1+2\gamma \eta \right){\theta }^{{{\prime}}{{\prime}}}+2\gamma {\theta }^{{\prime}}\right]+F{\theta }^{{\prime}}-{F}^{{\prime}}\theta =0,$$

15 $$\begin{aligned} \text{where }{C}_{3} =\frac{{k}_{hnf}}{{k}_{f}}\left\{\left[\left(1-{\phi }_{2}\right)\left\{\left(1-{\phi }_{1}\right)+{\phi }_{1}\frac{{\left(\rho {c}_{p}\right)}_{{s}_{1}}}{{\left(\rho {c}_{p}\right)}_{f}}\right\}\right]+{\phi }_{2}\frac{{\left(\rho {c}_{p}\right)}_{{s}_{2}}}{{\left(\rho {c}_{p}\right)}_{f}}\right\}\end{aligned}$$

the non-dimensional form of eq. 1 is

$$ \begin{array}{ll}    h=1-\frac{\epsilon }{2} (1+ {cos}{(4\pi \tilde x)}), &  \quad {-\frac{1}{4}<\tilde x< \frac{1}{4}}  \\   \quad    {= 1}   & \quad {Otherwise,}  \\   \end{array}  $$

The non-dimensional form of boundary conditions on stenosed artery are

16 $$\begin{aligned}F\left(0\right)&=0, \; { F}^{{\prime}}\left(0\right)=1, \; \theta \left(0\right)=1, \eta =0 \\  {F}^{{{\prime}}{{\prime}}}\left(\eta \right) &=0, \; {{\theta}^ {{\prime}}} \left(\eta \right)=0 \; at \; \eta =h.\end{aligned}$$

The non-dimensional parameters in above equations are Prandtl number $Pr={k}_{f}/{(\mu {C}_{p})}_{f},$ flow parameter is $\gamma =\sqrt{{\nu }_{f}{L}_{0}/{u}_{0}{R}^{2}}$ and concentration of Cu-Ag nanoparticles are presented by ${\phi }_{1}$ and ${\phi }_{2}$ respectively. The physical quantities i.e., heat transfer coefficient $N{u}_{x}$ and Skin friction coefficient ${C}_{f}$ of flow field.

17 $${C}_{f}=\frac{{\tau }_{w}}{\frac{1}{2}{\rho }_{f}{U}_{0}^{2}},$$

18 $$Nu=\frac{x{q}_{w}}{{k}_{f}({T}_{1}-{T}_{0 })},$$

We can find expression for shear stress ${\tau }_{w}$ as

19 $${\tau }_{w}={\mu }_{hnf}{\left.\frac{\partial u}{\partial r}\right|}_{r=R},$$

and heat flux is

20 $${q}_{w}={-k}_{hnf}{\left.\frac{\partial T}{\partial r}\right|}_{r=R},$$

Non-dimensional form of Eqs. (17–18) becomes

21 $$R{e}_{x}^{1/2}{C}_{f}=\frac{1}{{\left(1-{\phi }_{1}\right)}^{2.5}{\left(1-{\phi }_{2}\right)}^{2.5}}{F}^{{{\prime}}{{\prime}}}\left(0\right),$$

22 $$R{e}_{x}^{-1/2}N{u}_{x}=-\frac{{k}_{hnf}}{{k}_{f}}{\theta }^{{\prime}}\left(0\right),$$

where $R{e}_{x}^{-1/2}$ shows the Reynolds number.

## Graphical results and explanation

**Figure 2:**
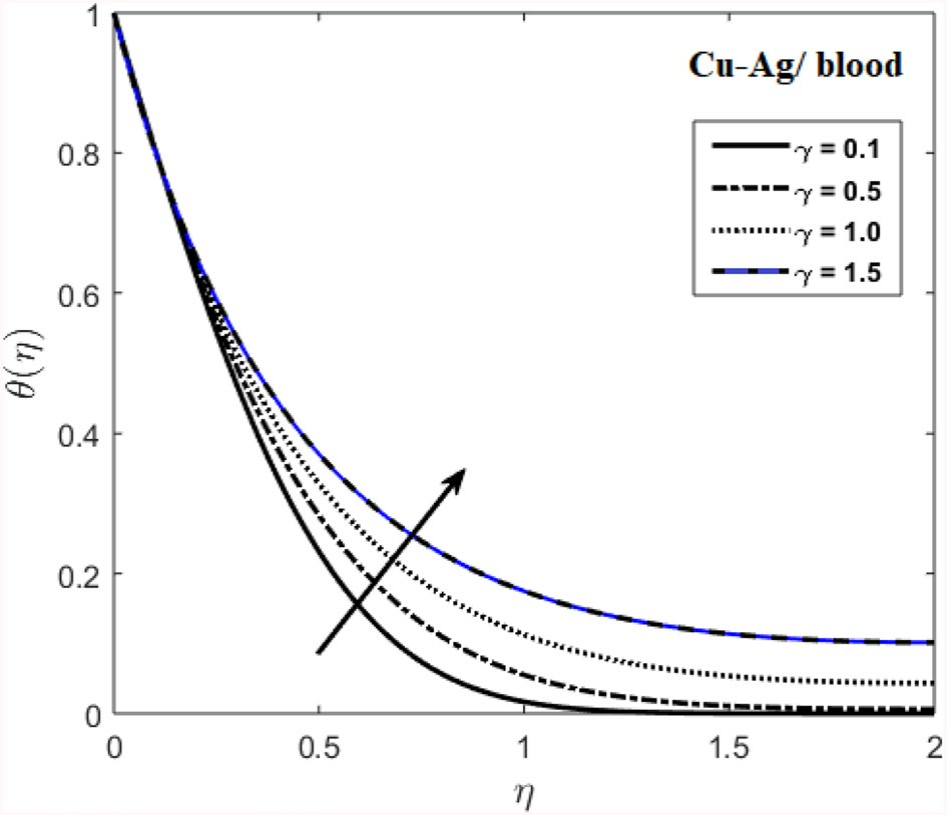
Behavior of flow parameter $(\gamma )$ on $\theta (\upeta$).

**Figure 3:**
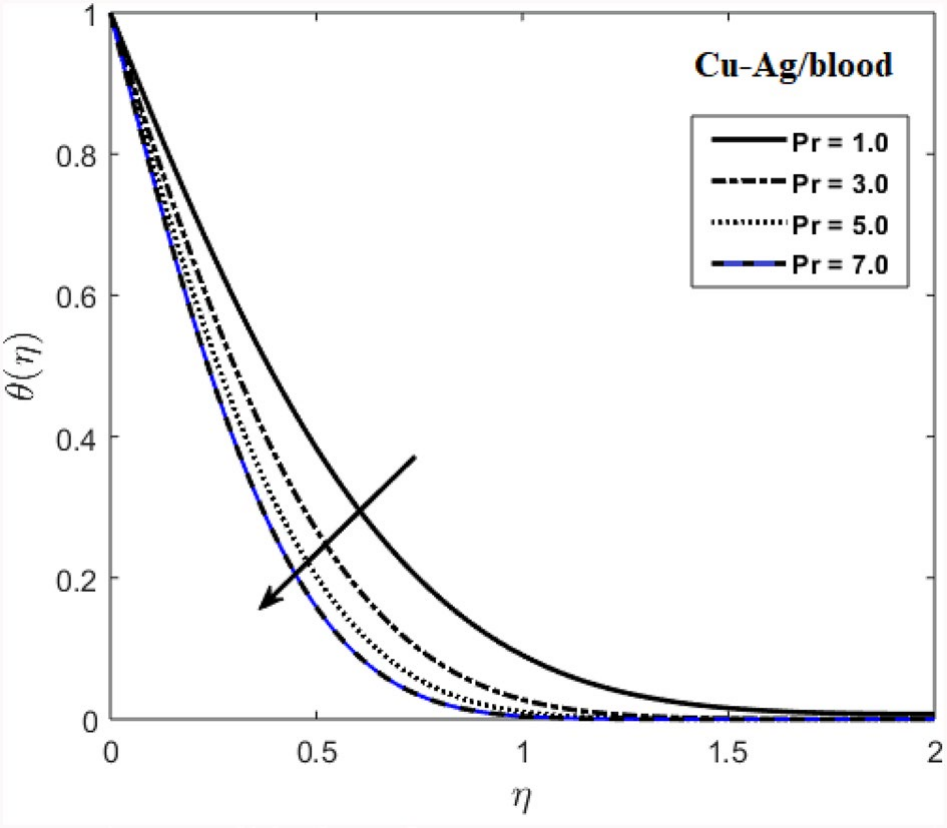
Distribution of $Pr$ on $\theta (\upeta$).

**Figure 4:**
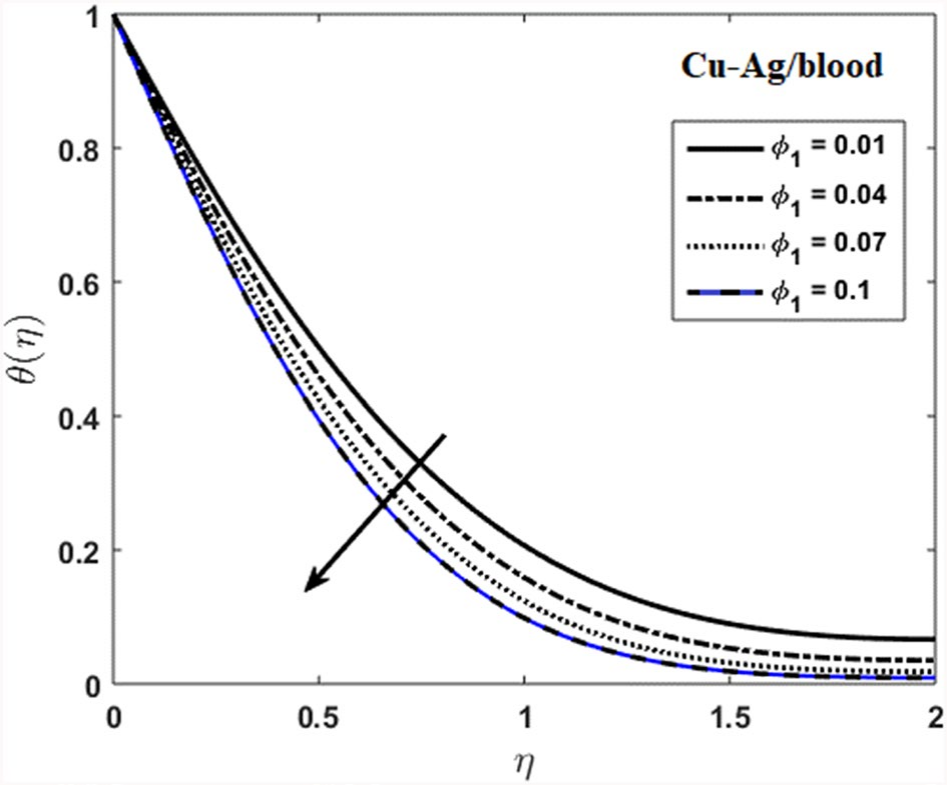
Consequences of nanoparticles volume fraction on $\theta (\upeta$).

**Figure 5:**
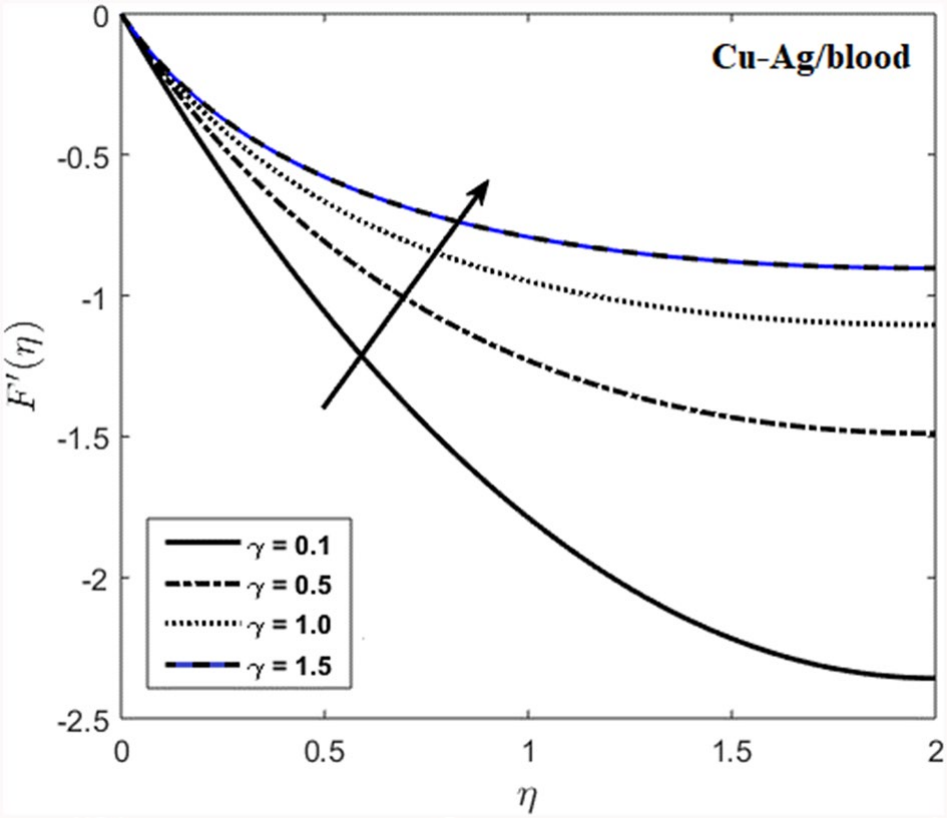
Behavior of flow parameter $(\gamma )$ on velocity.

**Figure 6:**
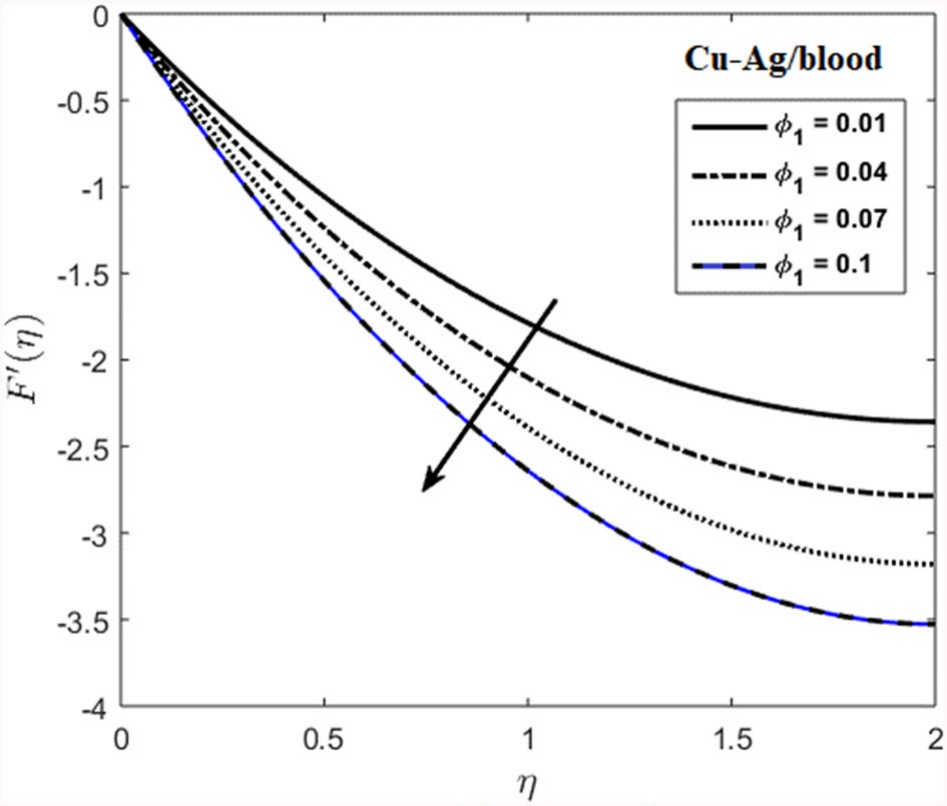
Effects of nanoparticles volume fraction on velocity.

**Figure 7:**
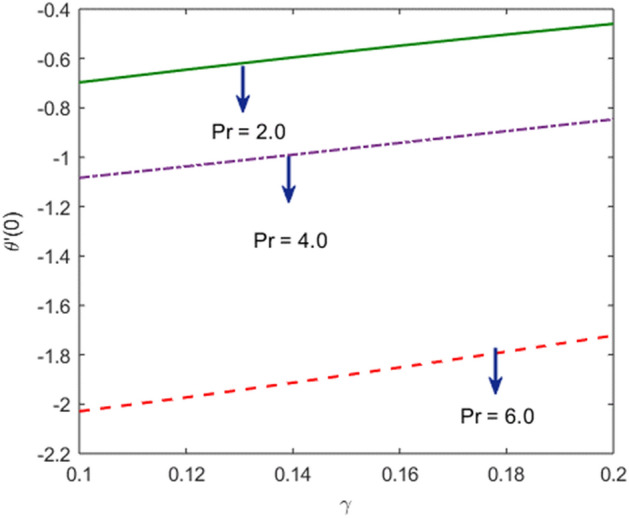
Consequences of $Pr$ and $\gamma$ on $-\theta {^{\prime}}(0$).

**Figure 8:**
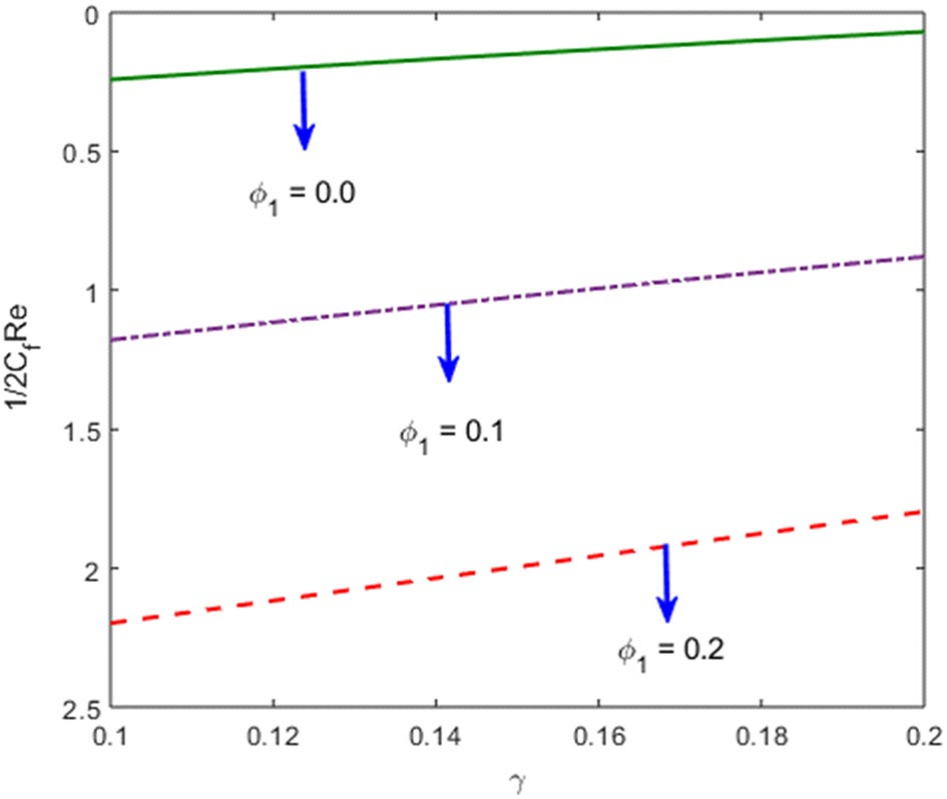
Variations of nanoparticle volume fraction and $\gamma$ on $\frac{1}{2}{C}_{f}Re$.

The effects of stenosed artery on the blood flow pattern and the consequences of various parameters are investigated. The graphical results for temperature and velocity are presented. The aim of this study is to obtain the best possible combination of physical and chemical properties in a unique fluid by using different materials. Figure 1 shows the geometry of constrict artery. The behavior of fluid flow parameter $\gamma$ on blood temperature is presented in Fig. 2. Temperature of blood goes by rising $\gamma$ values. Figure 3 shows the temperature results against $Pr$. The curve of temperature decreases by increasing $Pr$. The behavior of nanoparticles on temperature curve is shown in Fig. 4. Curve of temperature decreases due to increase in $\phi$. Basically the results in Figs.  2, 3, 4 reveals that temperature decreases with the addition of hybrid nanoparticles (copper, silver) for increasing values of $Pr$ and nanoparticles volume fraction and increases for $\gamma$. Hybrid nanoparticles shows remarkable properties which cannot be obtained by any component in individual state. The behavior of fluid flow parameter $\gamma$ on velocity curve is presented in Fig. 5. Velocity of blood enhance by rising $\gamma$. The consequences of nanoparticles on velocity of blood is presented in Fig. 6. Velocity of blood decreases by rising the values of $\phi_1$. This indicates that velocity is an decreasing function of nanoparticles volume fraction and this result may be useful for surgical doctors during surgery to put control on blood flow. Nusselt number variates due to change in Prandtl number values and nanoparticles volume fraction is shown in Fig. 7. Heat transfer coefficient curve goes down by rising the values of $Pr$ as it is a ratio between momentum and thermal diffusivity. Figure 8 shows the consequences of skin friction and the curve decreases gradually. Experimental values for solid nanoparticles Cu-Ag and base fluid (blood) are described in Table 1. Table 2 describes the results of $Pr$ and $\gamma$ on heat transfer. This table shows that the heat transfer coefficient values increases due to increment in $\gamma$ while decreases due to $Pr$. Table 3 presented the impact of $\phi$ and $\gamma$ on skin friction. Result shows that when $\gamma$ rises the values of Skin friction coefficient bending down and due to increment in $\phi$ solid nanoparticles volume fraction the values of Skin friction rises. All these properties and consequences of parameters may be helpful during operation procedures in tuning the flow of blood.

**Table 2:** Nusselt number $-\frac{{k}_{nf}}{{k}_{f}}\theta {^{\prime}}(0)$ with respect to $\gamma$ and Pr.

$\gamma$	Pr	$-\frac{{k}_{nf}}{{k}_{f}}\theta {^{\prime}}(0)$
0.1	2.0	− 0.24123
0.12		− 0.20305
0.14		− 0.16682
0.1		− 0.24123
	4.0	− 1.17916
	6.0	− 2.19785

**Table 3:** Values of $\frac{1}{2}{C}_{f}Re$ with respect to $\phi$ and $\gamma .$

$\gamma$	ϕ	$\frac{1}{2}{C}_{f}Re$
0.1	0.0	0.6970
0.12		0.6455
0.14		0.5959
0.1		0.6970
	0.1	1.0834
	0.2	2.0290

## Concluding remarks

In current article, the model studies the behavior of Prandtl number, flow parameter and nanoparticle volume fraction on temperature and velocity of blood. The hybrid nanofluid is considered as a mixture of blood and Cu-Ag NPs. Some main lists of the results can be summarized as follows:

1. Rising the values of $\gamma$ and the nanoparticle size intensifies the velocity and flow of blood.
2. Temperature of blood decreases due to increase in $Pr$, $\gamma$ and nanoparticle volume fraction values.
3. Heat transfer curve diminishes with the increase in $\gamma$ and $Pr$ values.
4. Skin friction curve declines by rising the values of nanoparticle volume fraction and $\gamma .$
5. The results indicate that addition of nanoparticles can help in improving flow of blood through arterial stenosis.

## List of symbols

R: Radius of constricted region
${R}_{o}$: Width of unobstructed area
$\lambda$: Maximum height of stenosis
${L}_{o}/2$: Length of stenosis
$$(u, v)$$: Velocity components
$$\left(r,x\right)$$: Coordinate components
${\upmu }_{hnf}$: Dynamic viscosity of hybrid nano-fluid
${\uprho }_{hnf}$: Density of hybrid nano-fluid
${\mathrm{k}}_{hnf}$: Thermal conductivity for hybrid nano-fluid
${\left(\uprho {C}_{p}\right)}_{hnf}$: Heat capacitance of hybrid nano-fluid
$${\rho }_{f}$$: Blood density
$${C}_{f}$$: Skin friction
$$Nu$$: Nusselt number
$$R{e}_{x}^{-1/2}$$: Reynolds number
$$T$$: Dimensional temperature
${\upmu }_{f}$: Dynamic viscosity of base fluid
${\mathrm{k}}_{f}$: Thermal conductivity for base fluid
${\phi }_{1}$, ${\phi }_{2}$: Nanoparticles volume fraction
${(\rho {C}_{p})}_{f}$: Heat capacitance of base fluid
${\left(\rho {C}_{p}\right)}_{{s}_{1}}, {(\rho {C}_{p})}_{2}$: Heat capacitance of nanoparticles
${k}_{{s}_{1}},{k}_{{s}_{2}}$: Thermal conductivity for nanoparticles
${\rho }_{{s}_{1}}$, ${\rho }_{{s}_{2}}$: Densities of solid nanoparticles
$$\psi $$: Stream function
${\nu }_{f}$: Kinematic viscosity of base fluid
$$\gamma $$: Flow parameter
$$\eta $$: Transformed coordinate
$$Pr$$: Prandtl number
${\tau }_{w}$: Wall shear stress
$${q}_{w}$$: Heat flux
$\theta$: Non-dimensional temperature
